# A Promising Aid to Therapy

**Published:** 1920-06-12

**Authors:** 


### COLLOIDS IN MEDICAL PRACTICE.

### A Promising Aid to Therapy.

The use of colloids in medicine is to some extent new, and certainly it is still largely experimental; but this form of therapy has within recent times been brought so repeatedly to the practitioner's notice—by a multitude of advertisements, if not by his own reading—that it is decidedly time that some co-ordinated advice upon the subject was provided. With this end in view we note that the *Prescriber* has devoted the major part of its current issue to a complete *résumé* of the subject, and it must in justice be said that a very fair and complete set of deductions are provided for the doctor's consideration.

Generally, the tone of these findings distinctly favours the fact that in administration of colloidal solutions we have new and valuable therapeutic means which, in some instances at least, mark a distinct advance upon old-time methods. There is one great difficulty at present experienced, and one, we believe, that largely accounts both for the failures experienced in many quarters and the large amount of reasonable and unreasonable criticism which has been deluged upon certain advocates of these agents. This is the relative rarity of any working knowledge of physical chemistry among the members of that section of the medical community who come into actual contact with patients. There are, of course, brilliant exceptions, but one refers to the profession in general. In administering the simplest remedial agent—even a cod-liver oil preparation—success or failure depends upon the thorough understanding of the effect we need to produce, of what the agent can be expected directly or indirectly to achieve, and to some extent at least how it may do it. The curious behaviour of colloidal solutions is still as a closed book to many who call them to their aid, and therefore it is time to scan the subject a little more closely.

Merely to read the somewhat excusably optimistic announcements which accompany these products when they leave the manufacturer's hands is a method with obvious limitations. In bringing a much more helpful light to bear upon the subject, our contemporary has rendered a most useful service; one, indeed, which a short note like the present cannot hope to emulate; but if our own references will produce in the hospital world some degree of reconsideration of a most important subject, our main object will be achieved.

### Colloidal Antiseptics.

The customary antiseptics of hospital life seem to be so soundly established—some surgeons at least are so satisfied with their results, that the introduction here of the new colloids may in any circumstances seem fated to considerable postponement; but let us keep an open mind upon the subject and review a few of the possibilities. It is an oft repeated list which requires for the ideal antiseptic that it shall be capable of (*a*) precipating or inactivating bacteria, (*b*) of being chemically a germicide, and (*c*) of being non-caustic and non-toxic. The limitations in one or other of these respects of all our present agents are generally known, as also the fact that recent developments, such as irrigational methods, etc., have really tended to exploit other factors to make up for the deficiencies of the actual antiseptic. Now from a theoretical point of view, there are colloids which can reproduce *all* the above requirements. Of their success in actual practice we must still partially reserve our judgment, but it is of interest to sketch the outline mechanism of their possible action. The physical chemist will explain that the bodies of bacteria, physically considered, are colloidal in character, and the same applies to part at least of the body fluids in which their life cycle is passed. Also the particles of a colloidal solution are individually electrically charged, either positively or negatively. We are told that, generally speaking, the majority of bacteria contain an excess of negatively charged colloid particles, and, picture the process as we will, when these negative charges are neutralised the activity and specific power of the micro-organisms is suppressed. Therefore the obvious way to achieve this result is to add positively charged colloid particles, *i.e.*, a positive sol.

Such is the theory, and at present we think it is only partially borne out in practice, or at least that we have yet to find the ideal positive sol. Nevertheless, it is apparently a fact that if we take a known antiseptic, such as mercuric chloride, and produce a colloidal solution of it, then there is a summation of antiseptic powers from both chemical and physico-chemical effects, so that to obtain a solution of satisfactory strength, lower actual concentration is required. In this way a number of practical objections to the chemical in the non-colloidal state are removed. A remarkable instance is provided in the case of colloidal metallic silver. As a germicide it has approximately the same power as mercuric chloride, but in this case the colloid is non-toxic, non-caustic, and in strengths at present used, non-irritating. Also we see it stated that laboratory animals intravenously injected with it become immune for a certain period to anthrax and diphtheria bacilli. Expense of production will possibly limit its use as a general hospital germicide, but we believe there is much to be said for its therapeutic use, and its properties prove that the idea of the colloidal general antiseptic is far from being a myth.

### Therapeutic Use.

Having previously touched upon the colloidal nature of body fluids and the potential power we appear to have of influencing them by means of electrical-charged colloidal particles, it is both fascinating and easy to drift into discussion of how and when we can expect them to act. Some

**Colloids in Medical Practice—(*continued*).**

observers claim much, some a good deal less, and we leave the reader to consult the writings of McDonagh, Stevenson, Searle, and others, and to form his own conclusions. We can from personal experience affirm that many excellent clinical results have been obtained in practically all branches of medicine. The apparent uncertainty in many cases has undoubtedly arisen from a certain amount of "rule of thumb" administration and the inevitable partial misunderstanding of a new subject. However, as the *Prescriber* rightly points out, the doctor must satisfy himself that he is using colloids of the right strength, those whose properties are the best understood, and also those which are known to be adapted for the special *route* employed, orally, intravenously, etc., as the case may be. Also these solutions must be reasonably permanent—"protected," as it is technically described. A colloid solution is a solution of an insoluble substance and readily reverts to the non-colloidal state, whereupon any special properties possessed previously through its colloid character are obviously and completely dispersed. In a great majority of cases this reversion amounts to precipitation, and at the present the only guarantee that this will not occur inside or outside the body rests with the reliability and good faith of the firm or laboratory which sends out these products. Very few indeed can be kept any length of time in the true colloidal state, but such difficulties are reasonably easy to surmount under modern manufacturing and supply conditions, and it seems probable that with colloid, as with vaccine therapy, a valuable future lies before it, provided both its power and its limitations are thoroughly appreciated. Any practitioner will be well repaid for devoting a few moments' true attention to its details.

